# Amino acids trigger down-regulation of superoxide via TORC pathway in the midgut of *Rhodnius prolixus*

**DOI:** 10.1042/BSR20160061

**Published:** 2016-04-15

**Authors:** Ana Caroline P. Gandara, José Henrique M. Oliveira, Rodrigo D. Nunes, Renata L.S. Goncalves, Felipe A. Dias, Fabio Hecht, Denise C. Fernandes, Fernando A. Genta, Francisco R.M. Laurindo, Marcus F. Oliveira, Pedro L. Oliveira

**Affiliations:** * Laboratório de Bioquímica de Artrópodes Hematófagos, Instituto de Bioquímica Médica Leopoldo De Meis, Programa de Biologia Molecular e Biotecnologia, Universidade Federal do Rio de Janeiro, Rio de Janeiro, 21941-590, Brazil; † Laboratório de Bioquímica de Resposta ao Estresse, Instituto de Bioquímica Médica Leopoldo de Meis, Programa de Biologia Molecular e Biotecnologia, Universidade Federal do Rio de Janeiro, Rio de Janeiro, 21941-590, Brazil; ‡ Laboratório de Inflamação e Metabolismo, Instituto Nacional de Ciência e Tecnologia de Biologia Estrutural e Bioimagem, Universidade Federal do Rio de Janeiro, Rio de Janeiro, 21941-590, Brazil; § Laboratório de Biologia Vascular, Instituto do Coração (InCor), Universidade de São Paulo, Escola de Medicina, São Paulo, 05403-900, Brazil; ║ Instituto Oswaldo Cruz, Fundação Oswaldo Cruz, Rio de Janeiro, 21040-360, Brazil; ¶ Instituto Nacional de Ciência e Tecnologia em Entomologia Molecular (INCT-EM), Brazil

**Keywords:** amino acids, haem, mitochondria, *Rhodnius prolixus*, ROS, TOR

## Abstract

Sensing incoming nutrients is an important and critical event for intestinal cells to sustain life of the whole organism. The TORC is a major protein complex involved in monitoring the nutritional status and is activated by elevated amino acid concentrations. An important feature of haematophagy is that huge amounts of blood are ingested in a single meal, which results in the release of large quantities of amino acids, together with the haemoglobin prosthetic group, haem, which decomposes hydroperoxides and propagates oxygen-derived free radicals. Our previous studies demonstrated that reactive oxygen species (ROS) levels were diminished in the mitochondria and midgut of the Dengue fever mosquito, *Aedes aegypti*, immediately after a blood meal. We proposed that this mechanism serves to avoid oxidative damage that would otherwise be induced by haem following a blood meal. Studies also performed in mosquitoes have shown that blood or amino acids controls protein synthesis through TORC activation. It was already proposed, in different models, a link between ROS and TOR, however, little is known about TOR signalling in insect midgut nor about the involvement of ROS in this pathway. Here, we studied the effect of a blood meal on ROS production in the midgut of *Rhodnius prolixus*. We observed that blood meal amino acids decreased ROS levels in the *R. prolixus* midgut immediately after feeding, via lowering mitochondrial superoxide production and involving the amino acid-sensing TORC pathway.

## Abstract (precis)

*Rhodnius prolixus* midgut redox balance is regulated by a signalling pathway involving amino-acids/TORC/mitochondrial ROS to protect the midgut from oxidative damage.

## INTRODUCTION

Sensing incoming nutrients is a major signalling event that shapes physiology of most cell types, but is particularly critical for intestinal cells in order to perform their role in providing nutrients to sustain life of the whole organism. The TORC pathway is one of the major pathways involved in monitoring the nutritional status of eukaryotic cells [1,2]. One of its proteins, TOR, is activated by elevated amino acid concentrations, through few known effectors, and is inhibited by rapamycin, a property that originated the protein name, ‘**t**arget **o**f **r**apamycin’ [3].

Blood-sucking has independently appeared several times over the course of animal evolution. An important feature of haematophagy is that huge amounts of blood are ingested in a single meal. This characteristic is exemplified by the Chagas Disease vector *Rhodnius prolixus,* which can ingest up to 10 times the volume of their pre-feeding weight in a single meal [4]. Haemoglobin is the main blood protein, corresponding to 60% of the total protein content in the blood, and it can reach concentrations as high as 150 mg/ml. Haemoglobin digestion in the midgut of insects results in the release of large quantities of amino acids, together with the haemoglobin prosthetic group, haem, which decomposes hydroperoxides and propagates oxygen-derived free radicals [5,6].

Therefore, we previously suggested that attenuating or preventing the haem-induced oxidative challenge following a blood meal is a major adaptation to haematophagy [7]. Oliveira et al. [8] demonstrated that reactive oxygen species (ROS) levels were diminished in the midgut of the Dengue fever mosquito, *Aedes aegypti,* immediately after a blood meal. This effect was attributed to the negative regulation of a hydrogen peroxide-producing enzyme, dual oxidase. We proposed that this mechanism serves to avoid oxidative damage that would otherwise be induced by haem following a blood meal. Studies also performed in mosquitoes have shown that blood or amino acids control protein synthesis through S6K phosphorylation in midgut, ovaries and fat body [9–11]. Phosphorylated S6K is a downstream marker of TORC activation, a conserved complex that enhances translation of mRNA transcripts in response to the availability of nutrients [1]. Links between ROS and TORC have already been shown in different model organisms [12–14]. However, in spite of these reports, little is known about TOR signalling in insect midgut in response to feeding, nor about the involvement of ROS in this pathway. Here, we studied the effect of a blood meal on ROS production in the midgut of *R. prolixus*. We observed that blood meal amino acids decreased ROS levels in the *R. prolixus* midgut immediately after feeding, via lowering mitochondrial superoxide production and involving the amino acid-sensing TORC pathway.

## EXPERIMENTAL

### Ethics statement

All animal care and experimental protocols were conducted in accordance with the guidelines of the Committee for Evaluation of Animal Use for Research (Federal University of Rio de Janeiro, CAUAP-UFRJ) and the NIH Guide for the Care and Use of Laboratory Animals (ISBN 0-309-05377-3). The protocols were approved by CAUAP-UFRJ under registry #IBQM155/13. Dedicated technicians in the animal facility at the Instituto de Bioquímica Médica Leopoldo de Meis (UFRJ) carried out all protocols related to rabbit husbandry under strict guidelines to ensure careful and consistent animal handling.

### Insects

The *R. prolixus* colony was maintained at 28°C and 70–80% relative humidity at the Instituto de Bioquímica Médica Leopoldo de Meis–UFRJ. The insects used in the present study were adult mated females, and they were fed rabbit blood at 3-week intervals.

### Artificial feedings

Unfed animals (25–30 days fasting) were fed the following meals using an artificial feeding apparatus at 37°C: rabbit blood, plasma (obtained from heparinized rabbit blood) or Tyrode's physiological solution (0.14 M NaCl, 3 mM KCl, 2 mM CaCl_2_, 1 mM MgCl_2_, 0.4 mM NaH_2_PO_4_, 0.1% glucose) containing 100 μM ATP. Tyrode's solution was supplemented with 20 μM hemin, 50 mg/ml haemoglobin (Sigma), 50 mg/ml bovine albumin (Sigma), 50 μM MitoTEMPO (Sigma) or an amino acids mixture as described in Hara et al. [15] (L-Arg, 84 mg/l; L-Cys, 48 mg/l; L-Glu, 584 mg/l; L-His, 42 mg/l; L-Ile, 105 mg/l; L-Leu, 105 mg/l; L-Lys, 146 mg/l; L-Met, 30 mg/l; L-Phe, 66 mg/l; L-Thr, 95 mg/l; L-Trp, 16 mg/l; L-Tyr, 72 mg/l; L-Val, 94 mg/l; L-Gln, 103.75 mg/l) [15].

### Homogenate preparation

Adult female midguts were homogenized in phosphate-buffered saline (PBS; 10 mM Na–phosphate, 0.15 M NaCl, pH 7.4) containing a general protease inhibitor cocktail (Sigma).

### Rapamycin injection

*R. prolixus* were injected with 1.5 μl of 1.1 μM rapamycin (Sigma) dissolved in DMSO/Tyrode 1 h before feeding (adapted from Ref. [9]). All insects were maintained at 28°C in a humid chamber then were fed. All other controls were injected with the same concentration of DMSO/Tyrode. For microscopy assays, the insects were injected with 7.5 μl of 1.1 μM rapamycin. Then, anterior midguts (AMs) were isolated and homogenized after 1 h.

### *Ex vivo* ROS and mitochondria microscopy assays

For microscopy and HPLC assays, the wings, legs and dorsal plaques were dissected from the animals, and the haemocoel was filled with fluorescent probes dissolved in L15 medium culture (Gibco) containing 5% (v/v) fetal bovine serum. The samples were incubated in the dark at 28°C.

Initially, to assess ROS levels, the midguts were incubated with a 50 μM solution of oxidant-sensitive fluorophore dihydroethidium (hydroethidine, DHE) (Invitrogen). After 20 min of incubation, the midguts were washed with 0.15 M NaCl (saline solution) and immediately transferred to a glass slide for fluorescence microscopy analysis. It worth mentioning that, even after the ingestion of the blood meal, gut content is always behind the epithelium, and therefore, there is no absorption of light by the gut content, as neither the light incident on the tissue, nor the light emitted by the epithelial cells pass through the gut lumen. Quantitative evaluation of fluorescence levels was performed by acquiring images under identical conditions using a 20× objective and 100 ms exposure time in each experiment. The images were acquired in a Zeiss Observer.Z1 with a Zeiss Axio Cam MrM Zeiss and the data were analysed using AxioVision version 4.8 software. The #15 filter set (excitation BP 546/12 nm; beam splitter FT 580 nm; emission LP 590 nm) was used for DHE labelling.

To specifically visualize the mitochondria, the AMs were incubated with 1 μM Mitotracker® Green FM (Invitrogen) for 30 min. After incubation with 1 μg/ml 4′,6-diamidino-2-phenyindole (DAPI) (Sigma) for 5 min, the tissues were washed with saline solution and immediately transferred to a glass slide for fluorescence microscopy analysis. Quantitative evaluation of fluorescence levels was performed by acquiring images under identical conditions using a 100× objective and 400 ms exposure time in each experiment. The images were acquired in a Zeiss Observer.Z1 with a Zeiss Axio Cam MrM Zeiss within the Apotome mode (Grid D) and the data were analysed using AxioVision version 4.8 software. The #10 filter set (excitation BP 450–490 nm; beam splitter FT 510 nm; emission BP 515–565 nm) was used for Mitotracker® Green FM and the #1 filter set (excitation BP 365/12 nm; beam splitter 395 nm; emission LP 397 nm) was used for DAPI staining.

To assess mitochondrial membrane potential, the AMs were incubated with 100 nM membrane potential-sensitive fluorophore tetramethylrhodamine methyl ester solution (TMRE) (Invitrogen) for 20 min. Some AMs were pre-incubated with 0.5 μM FCCP (carbonyl cyanide 4-(trifluoromethoxy) phenylhydrazone) (Sigma) for 10 min and then incubated with 100 nM TMRE containing 0.5 μM FCCP for 20 min. After incubation with 1 μg/ml 4′,6-diamidino-2-phenylindole (DAPI) (Sigma) for 5 min, the tissues were washed with saline solution and immediately transferred to a glass slide for fluorescence microscopy analysis.

To assess mitochondrial ROS levels, the AMs were incubated with 5 μM MitoSox (Invitrogen) for 10 min. After incubation with 1 μg/ml DAPI for 5 min, the tissues were washed with saline solution and transferred to a glass slide for fluorescence microscopy analysis. The images were acquired in an Olympus IX81 microscope and a CellR MT20E Imaging Station equipped with an IX2-UCB controller and an ORCAR2 C10600 CCD camera (Hammamatsu). Image processing was performed with the Xcellence RT version 1.2 Software. Optical slices (0.1 μm) were generated. A 545/569 nm excitation filter and a 581/625 nm emission filter were used for TMRE and MitoSox staining and a 323/390 nm excitation filter and a 442/466 nm emission filter for DAPI staining.

### HPLC analysis of DHE products

To provide specific quantitative assessment of intracellular ROS levels, we performed HPLC fractionation of DHE oxidation products, as previously described [16]. After incubation with 150 μM DHE, as described above, the midguts were opened with tweezers and washed in PBS to remove intestinal contents. Pools of three gut epithelia each were frozen in liquid N_2_, homogenized in 100% acetonitrile (500 μl), sonicated (5 cycles of 4 W for 15 s on ice) and centrifuged at 13 000 x ***g*** for 10 min. The resulting supernatant was dried under a vacuum (SpeedVac SVC 100 Savant), and the resulting pellet was stored at -70°C until use. The dried samples were resuspended in PBS containing 100 μM diethylenetriamine pentaacetic acid (DTPA) (Sigma) and injected into an HPLC system (Waters) equipped with a photodiode array (W2996) and fluorescence detectors (W2475). Chromatographic separation of DHE oxidation products was performed using a NovaPak C18 column (3.9 x 150 mm, 5 μm particle size) equilibrated in solution A (10% acetonitrile and 0.1% trifluoroacetic acid) at a flow rate of 0.4 ml/min. After sample injection, a 0–40% linear gradient of solution B (100% acetonitrile) was applied for 10 min, followed by 10 min of 40% solution B, 5 min of 100% solution B and 10 min of 100% solution A. The amount of DHE was measured by light absorption at 245 nm, and the DHE oxidation products, hydroxyethidium (EOH) and ethidium (E), were monitored by fluorescence detection with excitation at 510 nm and emission at 595 nm.

### Catalase activity

Forty microlitres of fresh midgut epithelia homogenates (two organs for blood-fed insects and four organs for unfed insects), prepared as described above, were immediately centrifuged for 2 min at 17 500 x ***g***, and the supernatants were added to a reaction medium containing PBS and 100 μM H_2_O_2_. H_2_O_2_ disappearance was monitored for 1 min at 240 nm, as previously described [17,18]. The remaining aliquot of the homogenate was assayed under the same conditions in the presence of 2 μl of 1 mg/ml aminotriazole (Sigma) to correct for catalase-independent H_2_O_2_ degradation. Specific activities were calculated as aminotriazole-sensitive H_2_O_2_ decomposition using a molar extinction coefficient of 0.0394 mM^−1^.

### Protein carbonyl levels

Carbonyl contents of the midgut epithelia were assayed according to the protocol described by Levine et al. [19]. After rapid washing in distilled water, pools of 30 tissues were homogenized in 2 ml of a 5% sulfosalicylic acid solution. The resulting homogenate was divided into three equal aliquots of 400 μl and centrifuged at 15 000 x ***g*** for 5 min at room temperature. The supernatant was discarded, and the pellets were resuspended in 0.5 ml of 2 M HCl (for the blank) or 500 μl of 10 mM 2,4-dinitrophenylhydrazine (DNPH) (Sigma) in 2 M HCl for the remaining two tubes. DNPH leads to the derivatization of carbonyl groups, resulting in the formation of a stable dinitrophenyl (DNP) hydrazone product. The samples were kept in the dark for 1 h at room temperature and subjected to vortex-mixing for 30 s every 10 min, followed by the addition of 0.5 ml of 20% trichloroacetic acid. The tubes were centrifuged at 15 000 x ***g*** for 5 min, the supernatant was discarded, and the pellets were washed three times with ethanol:ethyl acetate (1:1). The pellets were resuspended in 1 ml of 6 M guanidine hydrochloride and incubated for 15 min at 37°C until the mixture became a clear yellow solution. After centrifugation at 15 000 x ***g*** for 5 min, the supernatants were collected, and their absorbance was read at 380 nm in a spectrophotometer (Shimadzu UV-2550). The amount of carbonylated protein was calculated using the carbonyl molar extinction coefficient (22 mM^−1^) and the results were corrected for protein concentration.

### Citrate synthase (CS) activity

citrate synthase (CS) activity was assayed according to the method described by Hansen and Sidell [20]. Pools of two AMs each were homogenized in 200 μl of saline solution. After 2 min of decantation, 10 μl of supernatant were incubated with 7.5 mM Tris buffer (pH 8.0) containing 30 μM DTNB (5,5′-dithiobis(2-nitrobenzoic acid)) (Sigma) and 250 μM acetyl-CoA. After 2 min, oxaloacetate was added at a final concentration of 500 μM, and DTNB reduction was measured for 3 min at 412 nm. The specific activity was calculated using the reduced DTNB molar extinction coefficient (13.6 mM).

### Statistical analysis

All experiments were repeated at least twice. Statistical analyses were performed using Student's *t* test with a 95% confidence interval or using one-way analysis of variance (ANOVA), followed by Dunnett's multiple comparisons post test (GraphPad Prism).

## RESULTS

### Blood meal decreases ROS levels and oxidative stress markers in the midgut of *R. prolixus*

**Figure 1:**
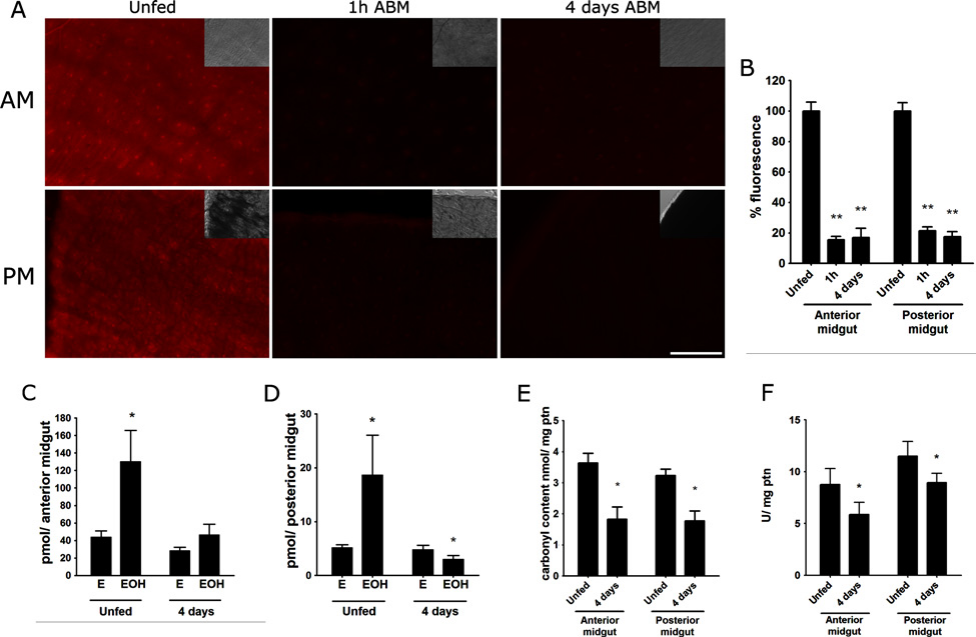
Blood meal decreases ROS levels and oxidative stress markers in the midgut of *R. prolixus* (**A**) Representative fluorescence microscopy images of the AM and posterior midgut (PM) after DHE staining. Midguts of unfed insects or insects dissected 1 h and 4 days after receiving a blood meal were incubated with 50 μM DHE. Scale bar represents 100 μm. The insets represent differential interference contrast (DIC) images. (**B**) Quantitative analysis of the fluorescence images shown in (A) (*n*=7–21 insects). **P<0.0001 (*t* test) compared with unfed. (**C** and **D**) HPLC fractionation of the DHE oxidation products, EOH and E, in the AM (**C**) and the PM (**D**) (*n*=6–8 pools). *P<0.05 (*t* test) compared with E. (**E**) Carbonyl content of the midgut epithelia was assayed as described in the Experimental section (*n*=3 pools). *P<0.05 (*t* test) compared with unfed. (**F**) Catalase activity in the midgut epithelia (*n*=10–16 pools). *P<0.05 (*t* test) compared with unfed. The data represent the mean ± S.E.M.

To test the effect of blood intake on ROS levels in the *R. prolixus* midgut, we incubated midguts from unfed insects and those taken 1 h and 4 days after blood meal (ABM) with the ROS-sensitive probe DHE. ROS levels were markedly reduced within 1 h ABM in both the anterior and posterior sections of the midgut (Figures 1A and 1B). ROS remained at similar levels during later stages of blood digestion, as determined by fluorescence images obtained at 4 days ABM (Figures 1A and 1B). To validate the results obtained using fluorescence microscopy, DHE oxidation products were analysed by HPLC, as DHE oxidation is known to yield two distinct products: EOH, selectively formed when DHE is oxidized by superoxide, and E, which can be generated through reactions with different oxidant species [21]. Figures 1(C) and 1(D) demonstrates that the EOH levels were remarkably higher in both midgut compartments of the unfed insects, indicating that, under this condition, superoxide radicals represent the dominant ROS produced by this tissue. Interestingly, at 4 days ABM, the only oxidant species that was reduced was EOH, as E levels remained unaltered in response to the blood meal. These data suggest that superoxide anion is the major ROS that is down-regulated in the digestive apparatus of blood-fed insects (Figures 1C and 1D). The reduced levels of ROS led us to measure carbonylated proteins, which are indicative of oxidative damage. Similarly, we measured catalase activity as a typical response to ROS production, as catalase gene expression is controlled by the redox-sensing NRF-2/Keap-1 signalling pathway, which is activated by increasing ROS levels [22]. Both oxidized protein levels and catalase activity were reduced in the midgut of blood-fed insects (Figures 1E and 1F).

### Role of mitochondria in midgut cells redox balance after a blood meal

**Figure 2:**
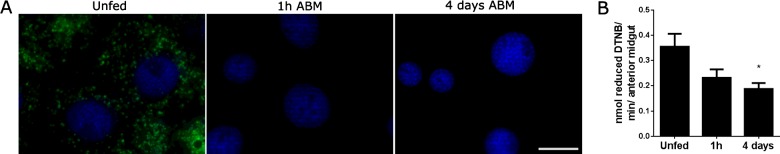
Blood meal reduces midgut mitochondrial density (**A**) AMs were dissected from unfed or blood-fed insects (1 h and 4 days ABM) and incubated with 1 μM Mitotracker® Green FM and 1 μg/ml DAPI, as described in the Experimental section. Scale represents 20 μm. (**B**) CS activity was immediately analysed in fresh homogenates from unfed or blood-fed insects (1 h and 4 days ABM). The assays were conducted as described in the Experimental section (*n*=8–12 pools). The data represent the mean ± S.E.M.; *P<0.05 (ANOVA, Dunnett's test, compared with unfed).

In addition to their role in energy metabolism, mitochondria are key players in redox homoeostasis and are an important source of cellular ROS [23]. When the mitochondrial probe Mitotracker® Green FM was used to gain insight into the dynamics of mitochondria in the midgut cells, we found that blood feeding dramatically decreased mitochondria content in the tissue (Figure 2A). To confirm this result, the activity of CS, a mitochondrial content marker enzyme [24] was assayed. The graph in Figure 2(B) shows that a blood meal led to a decrease in CS activity within 1 h and 4 days ABM.

**Figure 3:**
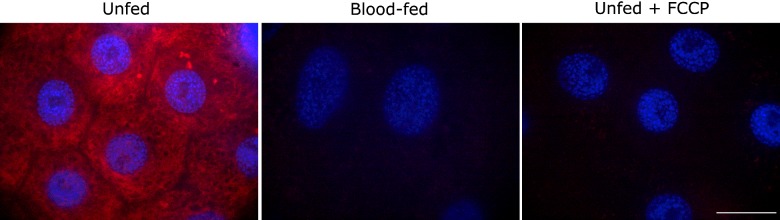
Blood meal reduces midgut mitochondrial membrane potential AMs were dissected from unfed, unfed with FCCP or 1 h blood-fed insects and incubated with 100 nM TMRE and 1 μg/ml DAPI, as described in the Experimental section. Scale represents 20 μm.

To evaluate the effect of a blood meal on mitochondrial membrane potential (*Ψ*_m_), which is a key mechanism involved in mitochondrial superoxide generation [25], AMs from unfed and blood-fed insects were incubated with TMRE, a *Ψ*_m_ sensitive probe [26], and fluorescence intensity was measured. Figure 3 demonstrates that blood feeding dramatically decreased *Ψ*_m_ in the midguts. Further analysis of tissues extracted from unfed insects treated with 0.5 μM FCCP confirmed that the change in TMRE fluorescence was primarily due to *Ψ*_m_.

**Figure 4:**
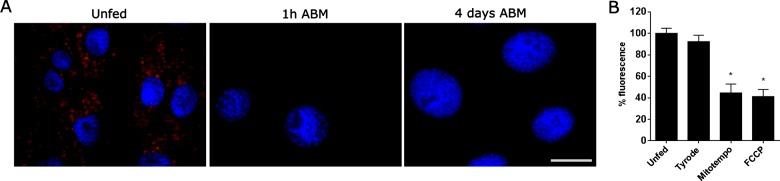
Mitochondria are a major feeding-responsive source of ROS in the midgut (**A**) AMs of unfed insects or insects dissected 1 h and 4 days after receiving a blood meal incubated with 5 μM MitoSox and 1 μg/ml DAPI, as described in the Experimental section. Scale represents 20 μm. (**B**) DHE oxidation in the AMs was evaluated by fluorescence microscopy. Starved insects were fed with Tyrode or 50 μM MitoTEMPO or the AMs were pre-incubated with 0.5 μM FCCP prior to incubation with DHE. The results represent the densitometric analysis of the images. The data represent the mean ± S.E.M. (*n*=5–32 insects). *P<0.0001 (ANOVA, Dunnett's test, compared with unfed).

Because a large fraction of ROS in the midgut could be attributed to superoxide production (Figures 1C and 1D), we tested whether the mitochondria were involved in this response. MitoSox staining (Figure 4A) indicated that blood-fed animals produced less mitochondrial ROS (mROS) than unfed insects. Therefore it seems that the majority of DHE signal in unfed insects comes from mitochondrial superoxide. This is reinforced by the graph in Figure 4(B) which shows that DHE oxidation was decreased in the midgut of insects after ingestion of the mitochondria-specific antioxidant MitoTEMPO [27] or after incubation with the proton ionophore FCCP. Overall, the data suggest that mitochondria produce higher ROS levels in unfed insects and a blood meal quickly reduces ROS production through a decrease in mitochondrial content and ∆*Ψ*_m_.

### Amino acids from blood proteins trigger down-regulation of ROS in the midgut via TORC pathway

**Figure 5:**
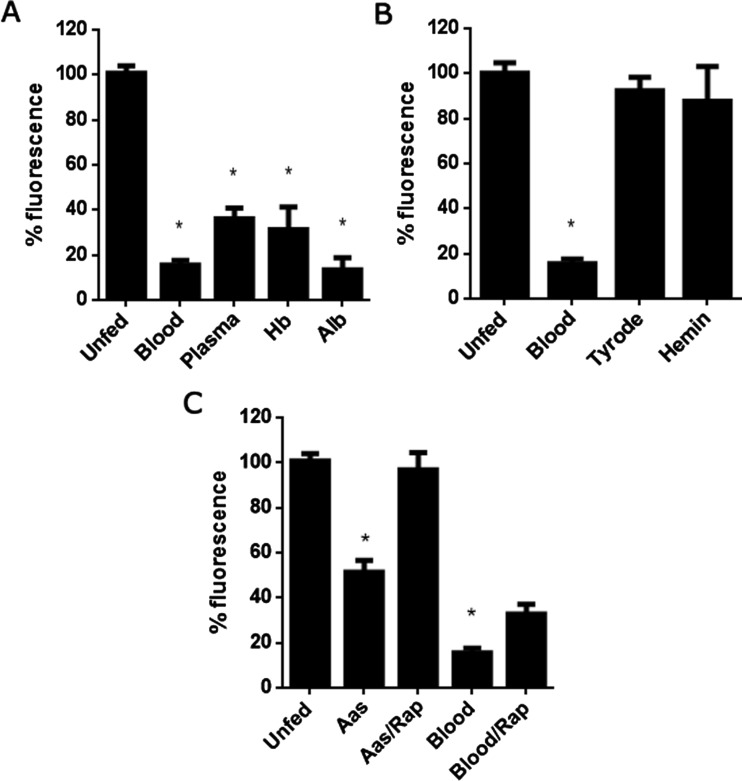
Amino acids from blood proteins trigger down-regulation of ROS in the midgut via TORC pathway DHE oxidation in the AM was evaluated by fluorescence microscopy. The results represent the densitometric analysis of the images. (**A**) Animals were fed the following meals: rabbit blood, plasma, 50 mg/mL haemoglobin or 50 mg/ml albumin. (**B**) Animals were fed with rabbit blood, Tyrode's solution or 20 μM hemin. (**C**) 1.5 pmol rapamycin were injected 2 h before feeding and animals were fed with amino acid mixture or blood. Quantitative analysis of the fluorescence images. The data represent the mean ± S.E.M. (*n*=4–32 insects). *P<0.0001 (ANOVA, Dunnett's test, compared with unfed).

To determine the signalling factor that triggers the decrease in ROS levels shown in Figure 1, unfed insects were fed with different meals and DHE fluorescence was evaluated in an *ex vivo* assay using a fluorescence microscope, 1 h after feeding. Blood plasma alone significantly decreased ROS levels (Figure 5A), an effect that was not observed when the insects were fed with Tyrode's physiological solution. Therefore, this solution was used as a vehicle to test the role of purified major blood components signalling for ROS down-regulation. The addition of haemoglobin in Tyrode's solution was able to lower ROS levels, but hemin alone was not effective (Figure 5B), differently from what happens in *A. aegypti*, where haem derived from haemoglobin is responsible for the reduction in midgut ROS levels after a blood meal [8]. However, albumin, a non-haem blood protein, was able to reduce DHE fluorescence, a result that led us to hypothesize that amino acids could be involved. Addition of an amino acid cocktail to the feeding solution markedly reduced DHE oxidation, and this effect was prevented by injection of rapamycin, an inhibitor of TOR (Figure 5C). Rapamycin injection in blood-fed animals partially prevented DHE oxidation, compared with unfed animals.

### TORC and mitochondrial ROS

**Figure 6:**
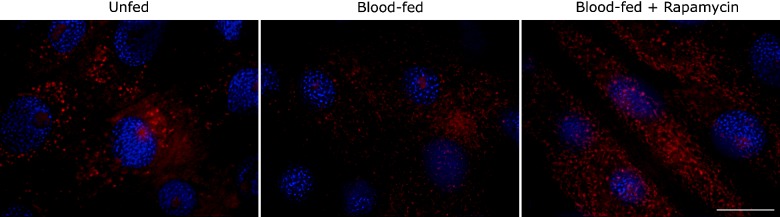
Rapamycin inhibits down-regulation of mitochondrial ROS, signalled by blood arrival in the midgut AMs of unfed insects or insects dissected 1 h after receiving a blood meal, were incubated with 5 μM MitoSox and 1 μg/ml DAPI, as described in the Experimental section. Rapamycin (7.5 pmol) was injected 2 h before blood feeding. Scale represents 20 μm.

It has been demonstrated in yeast and mammals that TORC activity influences mROS accumulation [12,13]. Therefore, we tried to establish a direct link between TORC pathway and mitochondria by injecting insects with rapamycin before blood meal and staining midgut tissue with MitoSox, a ROS-sensitive probe that is accumulated in the mitochondria. As shown in Figure 6, rapamycin prevented lowering of MitoSox signal signalled by blood arrival in the midgut, showing that the decrease in mROS that takes place after a blood meal depends on TORC signalling.

## DISCUSSION

ROS are produced in a wide range of physiological processes and mitochondria are not only responsible for energy transduction and ATP synthesis, but are also important regulators of redox balance [28,29]. As the actual capacity of haem to promote oxidative stress is linked to the functioning of ROS-generating cell metabolism [5,6], we studied here the effect of blood meal on redox balance in the midgut of *R. prolixus*.

Here, we demonstrated that superoxide production was markedly decreased in midgut cells immediately after blood ingestion (Figures 1A–1D). Decreased ROS levels in the gut are consistent with the observed low levels of carbonyl proteins and the induction of catalase activity, an antioxidant enzyme (Figures 1E and 1F). These results are in line with the hypothesis proposed for *A. aegypti* that some of the antioxidant mechanisms triggered by a blood meal are adaptations to prevent oxidative stress, rather than being a response to oxidative damage, which is the most common pattern found in the literature on free radical biology [8,30]. This finding suggests that this may be a general feature of haematophagous insects.

In a previous report in *A. aegypti* [30], it was shown that blood feeding caused a reversible reduction in mitochondrial oxygen consumption and H_2_O_2_ formation, an event that occurred in parallel to blood digestion. In *R. prolixus*, we show here that mitochondrial membrane potential decreased (Figure 3), together with a decrease in the mitochondrial content (Figure 2A), a conclusion that is reinforced by the observed reduction in CS activity (Figure 2B). An increase in electron supply to the respiratory chain, or proton-motive force, can increase the half-life of semi-ubiquinone radicals and drives one-electron reduction of molecular oxygen, forming superoxide. These data, together with the demonstration that mitochondria is a major source of ROS by MitoSox staining and that most of the ROS was suppressed by MitoTEMPO or FCCP, indicate that the decrease in ROS levels can be attributed to a decrease in membrane potential after a blood meal.

As shown in Figures 1(A) and 1(B), ROS reduction in the *R. prolixus* midgut occurs almost immediately, within 1 h ABM. In vertebrate endothelium, shear stress can regulate superoxide production [31], which led us to speculate that gut distention could play a role in the observed down-regulation of ROS. However, feeding insects with Tyrode's physiological solution did not alter DHE staining, excluding this possibility (Figure 5B). The addition of plasma, albumin or haemoglobin decreased ROS to a similar extent as a regular blood meal, suggesting that the delivery of nutrients is an important signal for ROS regulation in this insect midgut (Figure 5A) as already shown in *A. aegypti* [8].

Initially, we hypothesized that down-regulating ROS production might be an adaptive mechanism to prevent oxidative damage that otherwise arises from the massive accumulation of haem in the gut. However, in contrast with *A. aegypti* [8]*,* hemin did not decrease ROS levels in *R. prolixus* midgut (Figure 5B). mROS production is influenced by the cell energy balance and it was shown to stimulate insulin secretion in vertebrates [32]. Thus, it is conceivable that ROS production in *R. prolixus* midgut is involved in energy metabolism signalling, and not only as a potential source of oxidative stress.

Sensing of nutrients involving TORC is a conserved pathway from fungus to mammals, which regulates the protein synthesis and cell growth [3]. Hara et al. [15] showed, for the first time, that amino acid could activate this pathway. As expected in Figure 5(A), an amino acid mixture was capable of decreasing ROS levels similar to a blood meal. Rapamycin is an antifungal purified from *Streptomyces hygroscopicus* that has potent immunosuppressant and antiproliferative properties and its cellular target is the TOR complex [2,3,32]. Rapamycin injection opposed the effect of amino acids on ROS levels in the midgut, suggesting, for the first time, a cross-talk between TORC pathway and ROS regulation in insects (Figure 5C).

Few studies have already shown that TORC activity can be correlated with mitochondrial activity, regulating oxygen consumption, oxidative capacity, mROS accumulation and ROS sensing [12–14], being an important mechanism of longevity regulation in yeasts [33] and flies [34]. Low TOR signalling in flies fed with low protein diet, increased the activity of mitochondrial electron transport chain and produced more ROS, which induced a protective response at the organismal level through mitohormesis and increased longevity [34]. Rapamycin was capable to avoid mROS diminishing in the midgut of *R. prolixus* (Figure 6), signalled by blood arrival.

We show here that in unfed *R. prolixus* insects, high levels of mROS and carbonyl proteins were found in the gut, together with increased *Ψ*_m_, CS and catalase activities. Upon increased nutrient supply after ingestion of blood or amino acid-supplemented solution, mROS decreased along with mitochondria content, *Ψ*_m_ and oxidative stress markers.

Physiologically, these adaptations described in the hemipteran *R. prolixus* seem similar to what was reported for *A. aegypti*, where a dramatic reduction in ROS was also observed ABM [8]. However, as the blood-sucking habit arose independently in these two species, completely different mechanisms were recruited by evolutionary forces to fulfil a similar role: reduce midgut ROS generation upon blood meal by *i)* reducing mitochondrial membrane potential in *R. prolixus* and *ii)* reducing NADPH oxidase activity in *A. aegypti*. It is also relevant to note that these two distinct mechanisms that are acting in these two insect species are triggered by different signalling pathways. Although in the mosquito lowering of ROS levels depends on dietary haem activating a protein kinase C, in the triatomine bug down-regulation of mitochondrial ROS is under control of amino acids from the blood meal signalling through the TORC pathway.

We propose that blood meal ingestion signals to epithelial cells the input of nutrient, leading to activation of the TORC pathway, which will control mROS production to prevent oxidative damage. Taken together, data presented here highlight that nutrients can change mitochondrial dynamics, affecting redox equilibrium in the midgut, showing a direct link between TORC pathway and mitochondria. As superoxide is a major regulator of autophagy and its production can be induced by amino acid starvation in other organisms [35], further research will be needed to elucidate the molecular details of the cross-talk between mitochondrial signalling and the TORC pathway that drives the decrease in mitochondrial activity that takes place after a blood meal.

## Abbreviations

ABM: after blood meal
AM: anterior midgut
CS: citrate synthase
DHE: dihydroethidium
E: ethidium
EOH: hydroxyethidium
FCCP: carbonyl cyanide 4-(trifluoromethoxy) phenylhydrazone
mROS: mitochondrial ROS
PM: posterior midgut
ROS: reactive oxygen species
TMRE: tetramethylrhodamine methyl ester
